# Rule-Based Category Learning in Children: The Role of Age and Executive Functioning

**DOI:** 10.1371/journal.pone.0085316

**Published:** 2014-01-29

**Authors:** Rahel Rabi, John Paul Minda

**Affiliations:** Cognition and Perception Program, Department of Psychology, The University of Western Ontario, London, Ontario, Canada; University of Missouri-Kansas City, United States of America

## Abstract

Rule-based category learning was examined in 4–11 year-olds and adults. Participants were asked to learn a set of novel perceptual categories in a classification learning task. Categorization performance improved with age, with younger children showing the strongest rule-based deficit relative to older children and adults. Model-based analyses provided insight regarding the type of strategy being used to solve the categorization task, demonstrating that the use of the task appropriate strategy increased with age. When children and adults who identified the correct categorization rule were compared, the performance deficit was no longer evident. Executive functions were also measured. While both working memory and inhibitory control were related to rule-based categorization and improved with age, working memory specifically was found to marginally mediate the age-related improvements in categorization. When analyses focused only on the sample of children, results showed that working memory ability and inhibitory control were associated with categorization performance and strategy use. The current findings track changes in categorization performance across childhood, demonstrating at which points performance begins to mature and resemble that of adults. Additionally, findings highlight the potential role that working memory and inhibitory control may play in rule-based category learning.

## Introduction

Categorization is a fundamental decision-making process that allows us to meaningfully parse the world and group like objects together so that they can be treated equivalently. *Rule-based* categories are those in which the optimal rule is relatively easy to describe verbally [1]. Consider a category set in which round objects belong to one group and square objects belong to another group. These categories could be learned by applying the easy to verbalize rule: “category 1 objects are round”. However, even a simple classification rule like this requires sufficient cognitive resources, such as working memory and inhibitory control [2]. However, not all categories can be easily described by a verbal rule. *Non rule-based* categories are categories for which no easily verbalizable rule exists [3]. For example, consider a category in which most of the objects are small, most are round, and most are shiny. These objects share overall similarity with each other, but there is no single feature to act as the rule. Instead, these categories may be learned procedurally/implicitly by associating features with responses [4]. Both rule-based and non rule-based category learning has been studied in children [5]–[6], and adults [7]–[8]. Rule-based category learning is particularly interesting to study, because in our daily lives we encounter many instances where the information needed for making a classification decision is encapsulated in a rule. For example, when a child is learning to classify shapes they may apply categorization rules based on the number of sides the shape has. Similarly, when an adult is sorting laundry they may rely on rules to determine which clothes should be washed together. That is, they may apply rules based on clothing color: separating whites, darks, and lights into separate categories, or washing procedures: separating clothes that can be machine-washed from those that need to be dry-cleaned.

### Developmental Differences in Rule-Based Category Learning

Prior research has consistently demonstrated age-related improvements in rule-based category learning. For example, Minda, Desroches, and Church [9] compared categorization performance in 3-, 5-, and 8-year-olds, as well as adults. Results revealed that adults outperformed children on categories that were optimally learned by a disjunctive rule (e.g., 2 of the 3 stimulus features were relevant for the disjunctive rule). However, children could learn single-dimensional rules about as well as adults, suggesting that the ability to learn rules was not completely absent in children.

In addition to early childhood, developmental differences in the acquisition of category knowledge have also been examined in middle childhood [10]. Children between the ages of 8 and 12, as well as adults, learned several different category sets. In the rule-based category set, adults outperformed children because children persistently used the irrelevant dimension to make their category judgments, whereas adults were able to inhibit that dimension to their benefit. The fact that children persisted to use the irrelevant dimension as an imperfect rule implies that children lack the hypothesis testing abilities needed to find and use the optimal rule.

More recently, Visser and Raijmakers [11] used a task that required school-aged children (ages 4–13 years) and adults to categorize multi-dimensional stimuli that could be learned by adopting a rule-based or similarity-based strategy. Results showed no evidence of similarity-based representations occurring in children, suggesting that rule learning may be the default approach in children as well as adults. Additionally, many younger children did not complete the pre-training phase of the study, where participants learned to categorize single-dimensional stimuli and were required to obtain a certain number of correct classifications before they could progress to the test phase. The fact that younger children struggled to achieve criterion in pre-training reinforces the idea the rule-based categorization tends to improve with age.

### Rule-Based Categorization and Executive Functioning

The ability to exert control over thoughts and actions is a capacity often referred to as executive function. A key component of executive function is the use of explicit rules to guide behavior, which is an ability that develops gradually over the course of childhood. As children get older, they generally become increasingly skilled at using explicit rules to solve problems and categorize objects. Prior research has shown that developmental changes in rule use reflect the rate of development of the prefrontal cortex [12]. As such, we might expect age-related improvements in explicit rule-based categorization if cognitive development reflects, at least in part, an increase in prefrontal function. However, the topic of which executive processes are beneficial to rule-based category learning in children remains relatively unexplored. The goal of the current study was to examine how executive functioning is related to developmental changes in rule-based category learning.

Working memory and inhibitory control are two domains that have been tied to executive functioning and may prove important when learning rule-based categories. During rule-based categorization, working memory may be required to maintain and update rules that have been tested in memory, while inhibitory control may be required to inhibit incorrect rules. However, compared to adults, children have a reduced working memory capacity [13]–[14]. Visser et al. [11] suggested that one possible reason why younger children struggled with categorizing stimuli in their study might have been due to working memory limitations. In addition, compared to adults, children have reduced inhibitory control capacities [15]–[16]. Research by Huang-Pollock et al. [10] hinted at the role of inhibitory control in rule-based category learning, but inhibitory capacities were not measured in children in that study.

### Current Study

Although research has demonstrated age-related improvements in category learning, there has yet to be a thorough examination of the relationship between executive functioning ability and rule-based categorization performance in early and middle childhood. The current study was designed to examine differences between children's and adults' category learning abilities and strategies and how these differences were related to specific executive functioning abilities. Participants (children ages 4–11 years and adults) completed a rule-based categorization task, which required them to identify a single-dimensional rule. In order to correctly classify the stimuli, participants had to base responses on one dimension while ignoring irrelevant variation on another dimension.

Based on research described earlier demonstrating developmental variance in category learning, we predicted that categorization performance would improve with age. That is, since adults are presumed to have fully developed executive functioning abilities, they should be more successful at finding and using the correct rule to classify the stimuli compared to children. Executive functioning abilities continue to develop throughout childhood, and as a result younger children should be less effective at finding and using the correct rule compared to older children.

We were also interested in exploring the more direct relationship between category learning and executive functioning. Two key subcomponents of executive function (working memory and inhibitory control) continue to develop across childhood and may possibly be important for rule-based categorization [13], [15]. We measured working memory and inhibitory control abilities to identify which was most likely responsible for the influence of age on rule-based category learning in children and adults. That is, certain executive functioning abilities may help to explain why adults tend to outperform children on rule-based categorization. As well, exclusively among children, we were interested in examining whether executive functioning abilities would help to differentiate those children who adopted the correct rule-based strategy from those using a sub-optimal strategy.

## Methods

### Ethics Statement

The current study was approved by the University of Western Ontario Department of Psychology Research Ethics Board. Written informed consent was obtained from adult participants. Children provided verbal assent and parents also provided written consent prior to participation.

### Participants

Ninety-nine typically developing 4–11 year-olds were recruited through childcare centers and local schools (Table 1). Children were given stickers or pencils for participating in the study. Fifty-six adults (university students ages 18–27) were recruited from the Psychology research pool at the University of Western Ontario or through advertisement. Adults were given course credit or $10 for their participation in the study. Categorization performance did not differ between participants who received course credit and those who were paid.

**Table 1:** Description of Participants.

Age	Males∶Females	Mean Age in Years (SD)
4 to 5 year-olds (*n* = 18)	11∶7	5.42 (0.38)
6 to 7 year-olds (*n* = 24)	16∶8	6.55 (0.36)
8 to 9 year-olds (*n* = 38)	24∶14	8.98 (0.57)
10 to 11 year-olds (*n* = 19)	11∶8	10.48 (0.43)
Adults (*n* = 56)	27∶29	19.16 (1.64)

*Note.* Standard deviations are in parentheses. The groups consisted of 1 four year-old child, 17 five year-old children, 19 six year-old children, 5 seven year-old children, 20 eight year-old children, 18 nine year-old children, 16 ten year-old children, and 3 eleven year-old children.

### Measures

#### Categorization Task

**Figure 1:**
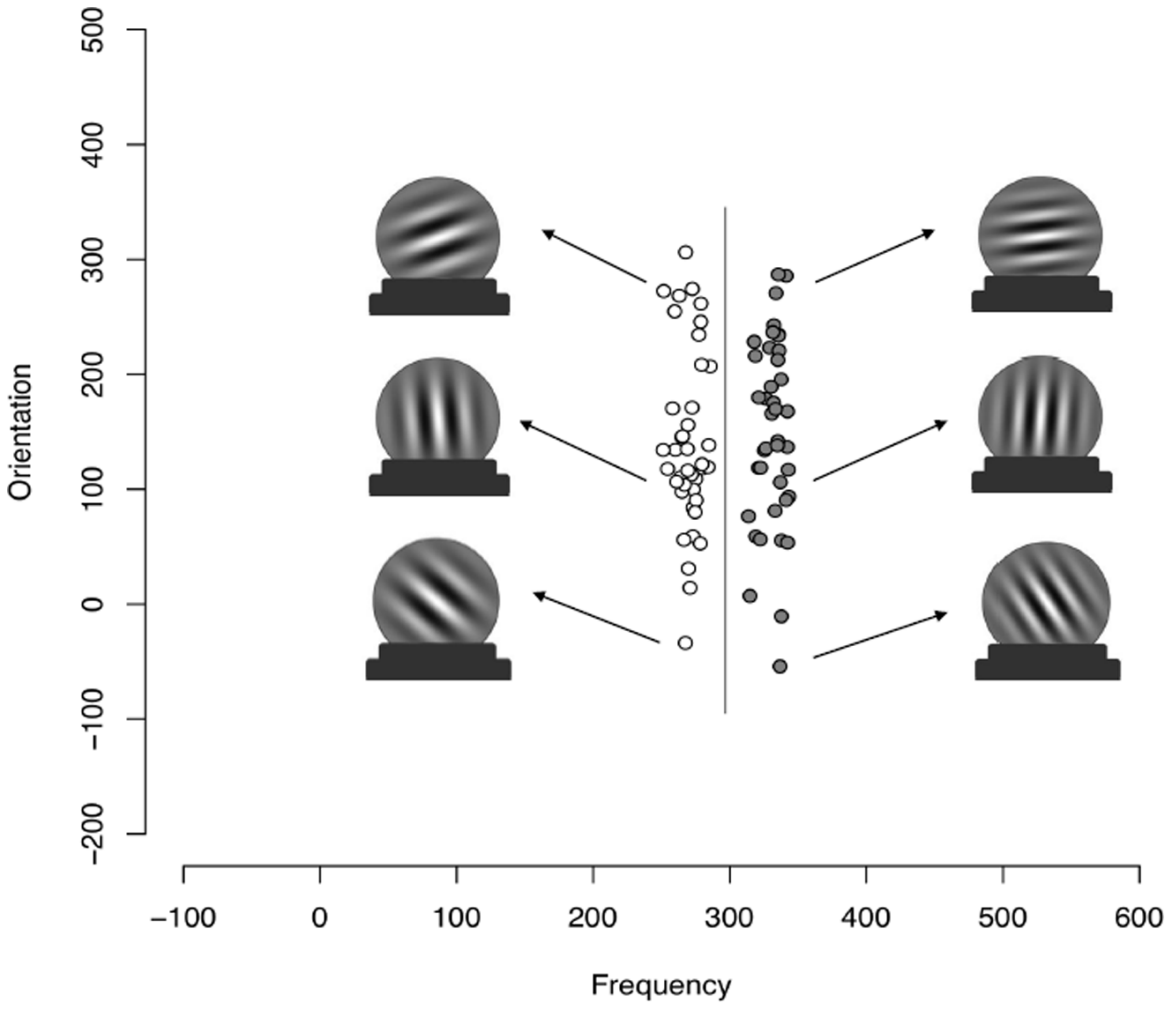
Rule-based category structure. The vertical line separating Category A and Category B represents the strategy that maximizes categorization accuracy (Ashby & Gott, 1988). Points on the left are members of Category A and points on the right are members of Category B. The learner must base responding on the frequency dimension while ignoring irrelevant variation on the orientation dimension. The optimal rule could be phrased as: “Crystal balls with few lines go in Category A, crystal balls with many lines go in Category B”.

Participants learned to classify sine-wave gratings that varied in spatial frequency and orientation (Figure 1). The full categorization task consisted of 80 stimuli. The presentation order of the 80 stimuli was randomly generated for each participant. We used the PsychoPy package [17] to generate a sine wave grating (a Gabor patch) corresponding to each coordinate sampled from a distribution [2], [18]. The distribution of each category was specified by a mean and variance for frequency and orientation, and covariance between them. For each category, 40 values were randomly sampled from a multivariate normal distribution described by the parameters for that category (Table 2). The sine wave grating frequency was calculated as *f* = .25+(χ_f_/50) and orientation was calculated as o = χ_o_×(π/500). Two solid bars were added to the bottom of each stimulus, so that the stimulus resembled a “crystal ball” which would then be classified as belonging to a certain wizard (category).

**Table 2:** Distribution Parameters for the Rule-Based Category Set.

	μ_f_	μ_o_	σ^2^_f_	σ^2^_o_	*cov_f, o_*
Category A	270	125	75	5000	0
Category B	330	125	75	5000	0

#### Forward Digit Span

Participants heard a recording of a two-digit number sequence at a rate of approximately one digit per second, and the participants were asked to repeat the sequence back to the experimenter in the same order [19]. The task began with four practice trials in which participants responded and received feedback. Children heard three sequences at each sequence length and as long as they repeated at least one of them correctly they continued on to the next sequence length, for a maximum length of ten digits. The task was over once the participant was unable to repeat any of the sequences at a given length. No feedback was given throughout the task. The forward digit span score was calculated as the total number of correct responses given.

#### Backward Digit Span

The procedure for the backward digit span was the same as that for the forward digit span except that the participant was required to recall the digits in reverse order so that the last number was said first and the first number was said last, for a maximum of eight digits. The task was scored as the total number of correct responses.

#### Flanker Task

A version of the Flanker task adapted from Botvinick, Nystrom, Fissel, Carter, and Cohen [20] was used. The experiment was built using REALbasic 5.1. A set of five arrows was presented in a row on the computer screen and participants were asked to indicate the direction of the central arrow (target). The target was flanked by two identical arrows on either side (distractors) that were either pointing in the same direction (congruent trial) or the opposite direction (incongruent trial) of the target arrow. Neutral trials were also included where arrows surrounding the target arrow were replaced by squares. The task consisted of 60 trials (20 congruent, 20 incongruent, and 20 neutral) presented in randomized order and displayed to all participants in the same sequence. Each stimulus was presented for 4000 ms with an interstimulus interval of 1000 ms. Prior to the experiment participants received five practice trials that were not analyzed. The difference in mean reaction time between correct responses on congruent and incongruent trials (i.e., a difference score) and number of task errors (i.e., incorrectly indicating the direction of the central arrow) was used as a measures of inhibitory control. Larger difference scores were indicative of less efficient interference control. The task lasted approximately five minutes.

#### Go/No-Go Task

The second inhibitory task presented was the Go/No-Go task [21]–[22]. The experiment was built using REALbasic 5.1. Participants were presented with four different stimuli: a red square, a blue square, a red circle, and a blue circle. In the first block the individual was instructed to press a button every time a square appeared on the computer screen, irrespective of its colour (go trial), but to make no response when a circle appeared (no-go trial). In the second block, the individual was instructed to press a button every time a blue figure (square or circle) appeared on the computer screen, but to make no response when a red figure appeared. The whole task consisted of 60 trials (30 stimuli presented per block and the blocks were counterbalanced) presented in randomized order and displayed to all participants in the same sequence. Thirty percent of the trials were no-go trials (i.e., 18/60 trials). Each stimulus was presented for 800 ms with an interstimulus interval of 2000 ms. Prior to the experiment participants received five practice trials that were not analyzed. The total number of commission errors (i.e., incorrectly responding to a no-go trial) was measured. The task lasted approximately five minutes.

#### Simon Task/Spatial Conflict Task

The third inhibitory task presented to participants was the Simon task [23]. The experiment was built and run using the Psychology Experiment Building Language (PEBL) software [24]. Participants were first presented with a fixation cross in the center of the screen that remained visible for 400 ms. Immediately after the cross had disappeared, participants were instructed to press the left key in response to the red circle or the right key in response to a blue circle as fast as possible, regardless of stimulus location. The timing began with the onset of the stimulus, and the response terminated the stimulus. The trials on which the stimulus location was on the same side as the required response were the congruent trials and the trials on which the stimulus location was on the opposite side of the required response were the incongruent trials. The whole task consisted of 70 trials (30 congruent trials, 30 incongruent trials, and 10 neutral trials) presented in randomized order to each participant. Prior to the experiment participants received five practice trials that were not analyzed. Similar to the Flanker task, difference scores were calculated by computing the difference in mean reaction time between correct responses on congruent and incongruent trials. Difference scores were used to control for large individual differences in speed of responding. Without such a subtraction, a high or low score could be attributed to the participant simply being a slow or fast responder. Task errors were also computed (i.e., pressing the incorrect key in response to the stimulus). The task lasted approximately five minutes.

### Procedure

#### Session 1: Category Learning & Working Memory Tasks

**Figure 2:**
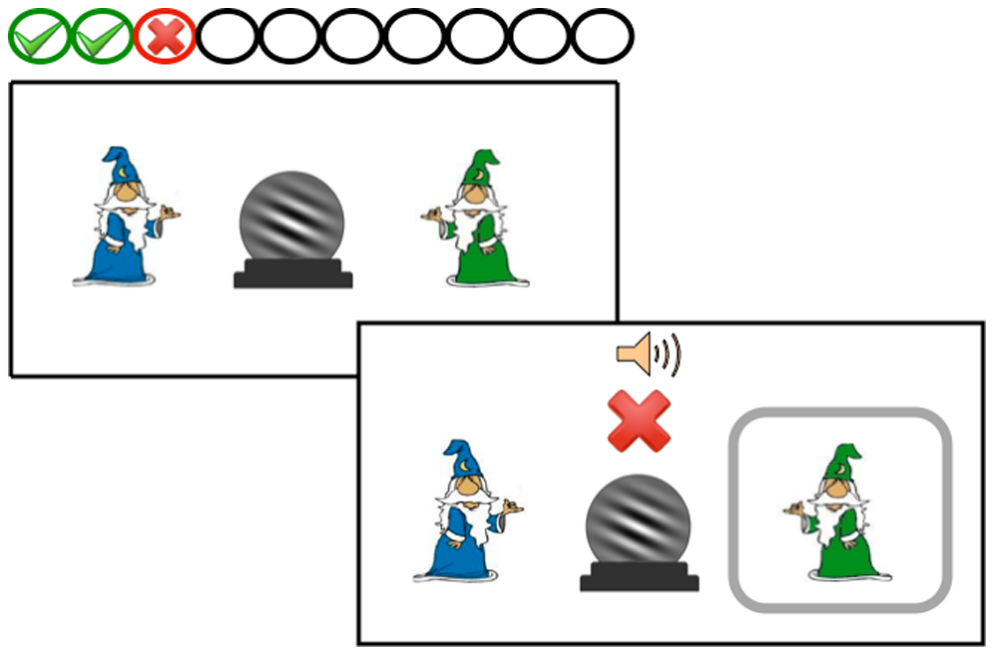
A sample trial from the rule-based categorization task.

Children were tested individually in a room near their classroom. The child and the experimenter were seated at a table in front of a 13-inch Apple MacBook computer. During the first testing session, children were told that they would be playing a game in which they would see pictures of crystal balls on the computer screen and that some of the crystal balls belonged to a blue wizard and some belonged to a green wizard. They were told that their job was to figure out which crystal balls belonged to the blue wizard and which belonged to the green wizard by clicking on the correct wizard on the screen (see Figure 2). On each trial, a picture of a crystal ball appeared in the middle of the screen and pictures of two “category labels” (blue or green wizard) were shown in the top left and right corners of the screen. The crystal ball remained on the screen throughout the entire trial. The correct category label was circled after each response regardless of whether the response was correct or incorrect. Correct responses were indicated with a bell sound and a green check mark displayed in the center of the screen for three seconds and incorrect responses were indicated with a red X for three seconds and a buzz sound. As well, a row of ten small white progress circles were shown along the top of the screen. Each time a trial was completed, a checkmark or X appeared in a circle at the top of the screen, depending on whether the child made a correct or incorrect response. After ten trials, when all the circles were filled, the circles all became white and a new set of ten trials began. These circles acted as a tool for subjects to keep track of their progress throughout the experiment. Following the rule-based categorization task, children received a short break, after which they were administered the forward and backward digit span. The first testing session lasted approximately half an hour.

#### Session 2: Inhibitory Control Tasks

Approximately 1–2 weeks after the categorization task and digit span tasks, each child's inhibitory control abilities were measured during a second testing session using three different computer tasks. First, participants completed the Flanker task on a 13-inch Apple MacBook computer. They were told that they would see an array of five arrows on the screen and their task was to press the arrow key on the keyboard that corresponded to the center arrow in the array as quickly as possible. Participants next completed the Go-No/Go task and were told that they would see a red circle, red square, blue circle, or blue square on the screen and their task was to press a button as quickly as they could every time a trial satisfied the rule given to them at the start of the task. After 30 trials, a new instruction screen appeared describing a new rule to follow. The last inhibition task administered to individuals was the Simon task. Participants were told that they would see a red circle or blue circle on the screen and their task was to press the “red circle key” every time they saw a red circle and the “blue circle key” every time they saw a blue circle as fast as they could.

The three inhibitory control tasks were always administered in the same order to all of the participants. The second testing session lasted approximately 20 minutes and children were given short breaks between inhibition tasks. Adults were tested individually using the same basic procedure as children except that adults were tested in a lab setting, whereas children were tested in a school setting (i.e., in an empty classroom). Adults completed each testing session on separate days, approximately 3–7 days apart. As well, adults read the instructions for each task on their own.

## Results

### Categorization Performance

**Figure 3:**
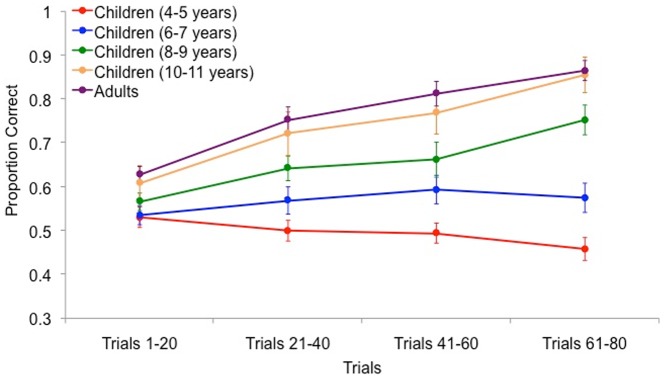
Category learning performance for children and adults across 80 trials. Error bars denote standard error of the mean.

The learning rate of the rule-based category set was examined in five groups of participants: children ages 4 to 5, 6 to 7, 8 to 9, 10 to 11, and adults. Of the participants who completed the categorization task, three adults were excluded from the analysis. One adult responded Category A to all trials and two adults displayed unusually and uniformly fast reaction times (1 participant responded between 100–300 ms on 53% of trials and the other responded between 100–300 ms on 76% of trials. The average categorization performance of both participants was below chance suggesting that they were not actively trying to solve the task). For each group of children and adults, the average proportion correct for each set of 20 trials was calculated. The resulting learning curves are shown in Figure 3 and demonstrate that while 8–9 year-olds, 10–11 year-olds, and adults showed evidence of category learning across trials, younger children (4–5 and 6–7 year-olds) struggled with the categorization task. A 5 (age)×4 (trial set) mixed ANOVA revealed a main effect for trial set, *F* (3, 441) = 22.69, *p*<.001, illustrating that learning occurred between the first and fourth sets of trials. As well, a main effect was also found for age, *F* (4, 147) = 18.86, *p*<.001, indicating that categorization performance differed across the different age groups of children and adults. Importantly, we found an interaction between age and trial set, *F* (12,441) = 4.98, *p*<.001, indicating that across the four sets of trials a difference emerged between the performance of children and adults.

A Tukey's HSD test was conducted to further examine this interaction and revealed that on the last set of trials, the categorization performance of 4–5 year-olds (*M* = .46, *SD* = .11) and 6–7 year-olds (*M* = .57, *SD* = .16) was significantly lower than 8–9 year-olds (*M* = .75, *SD* = .21), 10–11 year-olds (*M* = .85, *SD* = .18), and adults (*M* = .86, *SD* = .17), *p*≤.001 (in all cases). The final categorization performance of 8–9 year-olds did not differ from 10–11 year-olds (*p* = .22), but was significantly lower than that of adults (*p* = .02). Interestingly, there was no significant difference between final trial set performance among 10–11 year-olds and adults (*p* = 1.00), suggesting that 10–11 year-olds could perform at a similar level to adults.

### Model-Based Analysis

For insight into the response strategies used by children and adults, decision bound models were fit to each participant's data (see Maddox & Ashby [25] for a detailed description of these models). The models can be used to determine whether each participant is using a task appropriate strategy (i.e., basing responses on the frequency dimension) or a task inappropriate strategy (i.e., basing responses on the orientation dimension or guessing) to solve the task. We fit three different rule-based models to each participant's pattern of responses for the block of 80 trials. Two frequency strategies were fit to participant's responses. In one, the intercept of the decision bound and the noise parameter were allowed to vary (suboptimal frequency strategy). In the second, the intercept was set to the optimal value and the noise parameter was allowed to vary (optimal frequency strategy). A single orientation strategy was used, in which the intercept and noise parameter were allowed to vary. In addition, two guessing models were used which assume that the participant guessed or applied different strategies across trials within the block. One assumed that participants randomly responded *A* or *B* with equal probability for each response. This model had no free parameters. The other assumed that participants randomly responded *A* or *B* with unequal probability for each response. This model had one free parameter, the probability of responding *A*.

Parameters for each model were estimated using the maximum likelihood method [26]–[27], and in line with similar research conducted by Maddox et al. [28] and Visser & Raijmakers [11], the relative fit of the models was compared using the Bayesian Information Criterion (BIC, where *BIC* = *r* ln(*N*)−2 ln *L*; *r* is the number of free parameters, N is the number of trials being fit (80), and *L* is the likelihood of the model given the data) [29]–[30]. BIC is a measure of goodness of fit, which penalizes a model for extra free parameters. To find the best model to account for each participant's responses, a BIC value is computed for each model, and the model associated with the smallest BIC value is chosen.

### Model Frequencies

**Figure 4:**
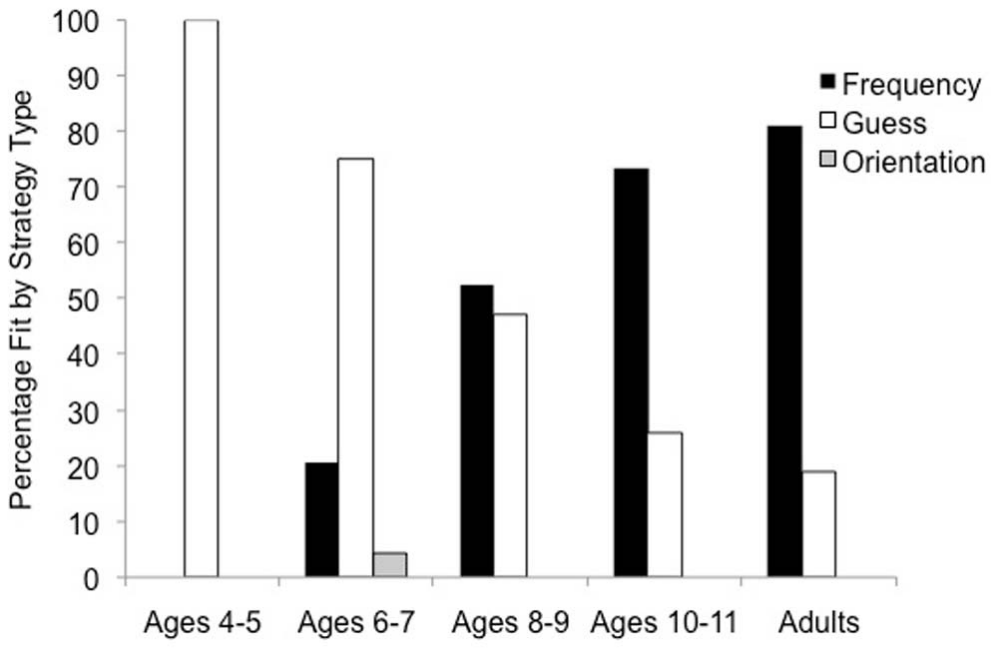
Percentage of participants fit by a frequency model (optimal or suboptimal), orientation model, or a guessing model. It should be noted that among those best fit by a frequency model, only two adults and two children (one 8-year old and one 10-year old) were better fit by the suboptimal frequency model.

The percentage of participants best fit by each model type was examined. As displayed in Figure 4, with each two-year increase in age, came an increased proportion of frequency rule users and a decreased proportion of guessers. Only one participant (a 6 year-old child) was best fit by the orientation model. Interestingly, no 4–5 year-olds were best fit by a frequency model, demonstrating that children at this age may find the rule-based categorization task difficult, impeding their ability to identify the correct strategy. It should be noted that children were tested based on their grade-level, and as a result of this, one 4 year-old was tested in the current study. Furthermore, it may only be concluded that 5 year-olds struggled with the task, since an insufficient number of 4 year-olds were tested. In comparison to 4–5 year-olds, a frequency model best fit 21% of 6–7 year-olds and 53% of 8–9 year-olds. Among 10–11 year-olds, a large proportion of children were identifying the task appropriate strategy (74%) and performance appeared to be more adult-like (81%). A χ^2^ -test that compared the proportion of frequency-users with those using a guessing strategy across 10–11 year-olds and adults was not significant [χ^2^(1) = .47, *p* = .49] suggesting that 10–11 year-olds were no less likely than adults to use the task appropriate strategy.

### Average Categorization Performance as a Function of Best Fitting Model

To examine whether children's general accuracy deficit in average categorization performance resulted from using a non-task appropriate decision strategy, we examined average categorization performance only for children (*n* = 39/99; five 6–7 year-olds; twenty 8–9 year-olds, and fourteen 10–11 year-olds) and adults (*n* = 43/53) who adopted the task appropriate strategy (i.e., a frequency based strategy). For those individuals using the task appropriate strategy, children (*M* = .78) and adults (*M* = .80) average categorization performance did not differ [*t*(80) = 0.93, *p* = .35], suggesting that those children using the task appropriate strategy performed at the same level as adults using the task appropriate strategy.

### Age-Related Categorization Improvements as a Function of Executive Functioning Abilities in Children and Adults

To investigate possible explanations for why adults tend to outperform children on rule-based categorization, a path analysis was conducted using Mplus version 5.1 [31] to examine whether executive functions play a role. Table 3 presents the means, standard deviations, and intercorrelations among variables when the data of all participants are considered. Age was correlated with all working memory and inhibition measures, and these executive functioning measures were all correlated with average categorization performance, hinting at a mediation relationship. A “working memory” composite score was created by combining forward and backward digit span scores (total digit span), with larger values indicating greater working memory. In addition, an “inhibitory control” composite score was created by combining Flanker and Simon difference scores, with smaller values indicating greater inhibitory control. Scores on these particular tasks were combined because they are both thought to tap into the same subtype of inhibitory control (i.e., interference control). Using this model, average categorization was regressed on working memory and inhibitory control, which were regressed on age. When overall fit of the data to the proposed model was examined, the fit of the model to the data was acceptable: non-significant χ^2^, TLI & CFI>.95, and RMSEA<.05. In the path analysis, both working memory (β = .73, *p*<.001) and inhibitory control (β = −.48, *p*<.001) were significantly associated with age. Average categorization performance was significantly associated with both age (β = .29, *p* = .009) and working memory (β = .21, *p* = .05). In contrast, average categorization performance was not significantly associated with inhibitory control (β = −.09, *p* = .26) in the sample of children and adults.

**Table 3:** Means, standard deviations, and intercorrelations among the study variables for all participants.

Variable	1	2	3	4	5	6	7
1. Age (months)	1.00						
2. Average Categorization Performance	.486**	1.00					
3. Forward Digit Span	.642**	.455**	1.00				
4. Backward Digit Span	.717**	.371**	.739**	1.00			
5. Flanker Difference Score	−.381**	−.254**	−.268**	−.267**	1.00		
6. Simon Difference Score	−.340**	−.191*	−.137	−.248**	.107	1.00	
7. Go/No-Go Commission Errors	−.512**	−.297**	−.381**	−.422*	.142*	.223**	1.00
Overall Mean	143.14	.67	15.33	6.96	106.21	67.69	2.47
SD	67.70	.16	3.49	3.30	92.52	65.92	2.33
Mean (children ages 4–5)	65.00	.50	12.33	3.5	95.85	139.63	3.38
SD	4.60	.05	2.09	1.72	75.91	112.02	2.28
Mean (children ages 6–7)	78.62	.57	13.00	4.75	169.55	71.59	4.46
SD	4.35	.10	2.75	1.75	111.91	71.98	3.06
Mean (children ages 8–9)	107.79	.66	14.92	6.39	136.74	72.76	2.79
SD	6.85	.15	2.23	2.14	101.06	50.13	1.63
Mean (children ages 10–11)	125.74	.74	15.05	7.05	103.24	60.66	2.68
SD	5.18	.16	2.78	2.34	110.14	50.57	2.21
Mean (adults)	230.64	.76	18.60	10.32	57.04	39.33	.85
SD	20.74	.14	3.14	2.86	22.40	32.88	1.16

* *p*<0.05;

** *p*<.01.

The path analysis revealed that when controlling for inhibitory control ability, a marginally significant indirect effect (β = .15, *SE* = .08, *p* = .057) was found between age and average categorization through working memory ability, indicating marginally significant mediation. This finding suggests that age-related improvements in categorization seen from childhood to adulthood may be partially explained by working memory ability. Since the direct association between age and average categorization was also significant (β = .29, *SE* = .11, *p* = .01), partial mediation was concluded. The mediated effect of working memory accounted for 30.86% of the total effect of age on average categorization. In contrast, when controlling for working memory ability, inhibitory control did not mediate the relationship between age and average categorization performance (*indirect effect* = .04, *SE* = .04, *p* = .27). In the inhibitory control composite score, a summed reaction time interference difference score was examined. In an effort to also examine accuracy on inhibition tasks, a second “inhibition errors” composite score was created by combining Flanker, Simon, and Go/No-Go errors. It should be noted that Flanker error score was correlated with Simon error score (*r* = .43) and Go/No-Go error score (*r* = .41), and Simon error score was correlated with Go/No-Go error score (*r* = .61), all at *p*<.001. Error scores on all 3 inhibition tasks were also correlated with age and average categorization, at *p*<.001. Inhibition errors did not mediate the relationship between age and average category learning (indirect effect = .07, *SE* = .05, *p* = .14).

### Relationship between Executive Functioning & Categorization Performance/Strategy Use in Children

To more carefully examine each component of executive functioning, additional analyses were conducted focusing on children's performance on the various task measures. Since adults are thought to have a fully developed executive functioning system, we were particularly interested in examining the relationship between category learning and working memory/inhibitory control (as measured by specific tasks) in children and how this relationship varied as a function of strategy use. This relationship was examined in 81 children ages 6–11. Four to five year-olds were excluded from this analysis, since earlier findings revealed that no children in this age range were capable of learning the correct rule in the categorization task. Age was correlated with all measures of working memory and inhibitory control, except for Simon difference score performance, suggesting that as children get older their executive functioning improves. Of particular interest, forward digit span (*r* = .27, *p* = .01) and flanker difference score (*r* = −.22, *p* = .02) correlated with average categorization performance among children. There was also a marginally significant correlation between average categorization performance and Flanker/Simon errors (both *r* = −.17, *p* = .06). When age was controlled for, partial correlations revealed that the relationship between forward digit span and average categorization remained significant (*r* = .18, *p* = .05) and the relationship between Flanker difference score and average categorization became marginally significant (*r* = −.15, *p* = .08). These results suggest that among the children, better forward digit span and Flanker performance were associated with higher average categorization performance. However, backward digit span, Simon, and Go/No-Go task performance did not correlate with average categorization performance.

Next, we examined whether children's age differed across best fitting strategy (task appropriate or task inappropriate). Age differed significantly across participant's whose data was best fit by a task appropriate (9.13 years) or task inappropriate (8.12 years) strategy [*t*(79) = 3.1, *p* = .01] suggesting that the older the children were, the more likely they would be using a task appropriate strategy. Next, we were interested in determining whether executive functioning differed between children who showed clear evidence of using the correct strategy, and those children who struggled with adopting the correct strategy. The executive function performance averages for children using the task appropriate and inappropriate strategy is displayed in Table 4. When looking at children's performance on individual task measures, forward digit span and backward digit span were significantly larger (implying better working memory) for those children best fit by a task appropriate strategy than by a task inappropriate strategy, [*t*(79) = 2.80, *p* = .006] and [*t*(79) = 2.30, *p* = .02] respectively. Among the inhibition tasks, the difference between strategy users was also significant for the number of Flanker errors [*t*(79) = 2.33, *p* = .02], and marginally significant for Flanker difference score [*t*(79) = 1.86, *p* = .06], suggesting better interference control among appropriate strategy users on the Flanker task. The difference between strategy users was marginally significant for the number of Simon errors [*t*(79) = 1.88, *p* = .06]. Simon difference scores and Go/No-Go commission errors did not differ as a function of strategy.

**Table 4:** Average executive functioning test performance as a function of task appropriateness in children (ages 6–11).

Measure	Task appropriate	Task inappropriate
Forward Digit Span	15.21	13.62**
Backward Digit Span	6.64	5.52*
Flanker Difference Score	115.77	159.80^m^
Flanker Errors	3.41	7.00*
Simon Difference Score	79.44	60.41
Simon Errors	6.33	8.62^m^
Go/No-Go Commission Errors	2.97	3.52

*Note*.

m *p* = 0.06,

* *p*<0.05;

** *p*<0.01 = significant t-tests across task appropriate and inappropriate values.

## Discussion

Previous research has outlined age-related differences in rule-based category learning but has yet to directly investigate the link between categorization performance and specific types of executive functions in children. In an extension of past research on rule-based category learning in childhood, the current study examined category learning, inhibitory control, and working memory in young children, middle-school children, and adults. As predicted, there was developmental variance in the acquisition of rule-based category knowledge in that categorization performance improved with age. Model based analyses provided important information regarding the strategy use of participants, which cannot be acquired from examination of performance accuracy alone. That is, the use of the task appropriate strategy increased with age. While 4–5 year-olds were not able to identify the task appropriate strategy, by age ten, children were as likely as adults to identify the correct strategy. Furthermore, it appears that earlier in childhood, children relied on guessing quite frequently when completing the categorization task, suggesting that they struggled with identifying the correct rule or did not apply a rule consistently. By age ten, children performed similarly to adults, suggesting that children at this age are able to identify and use categorization rules quite well. Interestingly, when directly comparing children and adults who used the task appropriate strategy, we found no rule-based performance deficit. In terms of executive functioning abilities, we found that age-related improvements in category learning were marginally mediated by working memory ability but not inhibitory control ability in children and adults. While as a group, children showed rule-based deficits compared to adults, a subset of children did have high categorization accuracy and were able to adopt the correct rule-based strategy. Among children, better categorization performance was associated with larger forward digit span score and marginally higher Flanker difference scores. When comparing children who used the task appropriate strategy with those using a task inappropriate strategy, we found that children who used the correct strategy were also those with higher forward and backward digit span scores, better Flanker performance, and fewer Simon task errors.

### Rule-Based Category Learning in Childhood

Our data is in line with previous developmental research showing that with age come improvements in rule-based category learning. This is likely a result of improvements in executive functioning that occurs with maturation. Minda, Desroches, and Church [9] showed that children can learn simple, single-dimensional rules at the same level as adults, but adults often outperform children on categories that are optimally learned by a disjunctive rule. The results from Minda et al. are consistent with the findings of the current study, in that children showed evidence of single-dimensional rule learning. The present findings illustrate that rule-based category learning ability gradually improves with age. Additionally, Huang-Pollock and colleagues [10] found that adults outperformed children (ages 8 to 12) on a rule-based categorization task because children persistently allowed the irrelevant dimension to guide their categorization judgments. In contrast, adults were able to inhibit the irrelevant dimension to their benefit. The model-based findings from our study are in line with those of Huang-Pollock et al., in that adults did on average, outperform children and adopt the task appropriate strategy more frequently than children. However, in contrast to the Huang-Pollock et al.'s findings, we found that younger children were better fit by a guessing model, rather than a model based on using the irrelevant dimension to make categorization decisions (i.e., orientation model). Our findings are comparable to those of Visser and Raijmakers [11], who found that the proportion of children classified as guessers decreased with age. Visser and Raijmakers interpreted such findings as indicating that the younger the children were, the greater the likelihood that they had an inconsistent response pattern. This suggests a growing ability in rule execution with age. One reason for the lack of participants fit by the orientation sub-optimal rule model could be due to the number of trials we used in our study. In order to accommodate the fact that we were testing young children with limited attention spans, our categorization task consisted of one block of 80 trials. Past studies testing the categorization performance of older children have typically included a larger number of trials (e.g., Huang-Pollock et al. included 400 trials in their study). Given more trials to complete, we predict that children may have relied on a sub-optimal rule more frequently then what we found in our study.

At the same time, we extend previous developmental research on category learning by showing how categorization performance and strategy use changes from early to middle childhood. While younger children (ages 4–7) had relatively low categorization accuracy, older children (ages 8–11) and adults showed higher categorization accuracy across trials. In terms of strategy use, children under the age of 9, tended to rely on guessing quite frequently to categorize the stimuli. However, between the ages of 10–11, children were as likely as adults to adopt the appropriate rule-based strategy.

### Executive Functioning & Category Learning

In an effort to explain age-related improvements in category learning, we examined whether executive functions (working memory and inhibitory control) would mediate the relationship between age and average categorization performance in children and adults. Results revealed a marginally significant partial mediating role of working memory. Given that the mediation was approaching significance, this may suggest that with age come improvements in working memory capacity, which in turn lead to improvements in rule-based categorization performance. However, additional research using various types of working memory tasks is needed to provide further support for such a conclusion. Past research is in line with our results demonstrating that working memory capacity tends to improve with age [13] and working memory is a primary determinate of categorization performance [32]. Furthermore, given the finding that the prefrontal cortex and working memory are still developing in children [12], [33], this may assist in explaining why children tended to perform more poorly relative to adults on the categorization task. In line with the findings of the current study, Minda et al. [9] demonstrated that while children showed poorer performance relative to adults in learning disjunctive, rule-based categories, increasing task demands for adults (i.e., having them complete a concurrent task that taxed working memory) actually resulted in child-like performance. Additionally, decreasing the task demands for children resulted in more adult-like performance. Previous research has likewise demonstrated that when adults perform a concurrent task that requires working memory during category learning, their rule-based categorization performance is impaired [34], [2], [35]. While prior research has suggested that age-related improvements in categorization may be partially explained by working memory capacity, the current study further tested this relationship by directly measuring working memory abilities in children and adults. In contrast, inhibitory control did not mediate the relationship between age and category learning. This suggests that age-related improvements in rule-based category learning could not be explained by improvements in inhibitory control abilities in children and adults.

In addition to investigating the mediating role of executive functions, we were also interested in examining whether differences in executive functioning abilities were associated with accuracy and strategy use among children. Given our earlier finding that the rule-based deficit diminishes when we focused exclusively on children and adults who learned the task appropriate strategy, it is evident that some children could learn the correct categorization rule quite well. Furthermore, even though executive functioning abilities are still developing across childhood, some children had sufficient executive functions required to search for and apply the correct rule. This is thought to be the case because while executive functioning continues to mature with age, rapid changes and developmental milestones occur early in childhood, making the relationship between age and rule-based category learning more complex [12]. Among children, enhanced categorization performance was associated with larger forward digit span scores and marginally higher Flanker difference scores. In terms of strategy use, results revealed that children who used the correct strategy were also those with higher forward and backward digit span scores, better overall Flanker performance (smaller difference score and less errors), and fewer Simon task errors. These findings are in line with research by Maddox and colleagues [28] showing that older adults were generally less accurate than younger adults on a rule-based categorization task. However, when the analyses focused only on participants who use the task appropriate strategy, the age-related rule-base deficit disappeared. Additionally, Maddox et al. found that the use of the task appropriate strategy on the rule-based categorization task was associated with better working memory performance (as measured by the total digit span). Interestingly, among the inhibition task measures, Stroop performance but not Wisconsin Card Sorting performance, was better among older adults best fit by the task appropriate strategy than by a task inappropriate strategy. Furthermore, these findings suggest that while the cognitive processes associated with working memory and inhibitory control are relevant to rule-based category learning, more research is required using a larger array of executive functioning tasks. It should be noted that among the inhibition tasks, only certain measures of inhibition performance were associated with strategy use in the current study. More specifically, while better overall Flanker performance and fewer Simon task errors were associated with task appropriate strategy use in children, Simon difference score and Go/No-Go task performance did not differ between strategy users. Future research may also benefit from examining whether certain subtypes of inhibitory control (i.e., interference control vs. inhibition of a prepotent motor response) are more related to rule-based category learning than other subtypes.

### Limitations & Future Directions

Results of the present study revealed that 4–5 year-olds struggled with the categorization task. In order to better examine the developmental trajectory of rule-based category learning, future research may benefit from using a different type of categorization task. Minda and colleagues [9] used a single-dimensional rule-based category set originally created by Shepard, Hovland, and Jenkins [36] in their study, and results showed that young children could learn these categories. In the category set used, perfect performance could be attained by the formation of a straightforward verbal rule (e.g., *if* black *then* Category 1). Young children were able to learn this category set because the rule was simple, easy to describe, and directly related to perception.

Additionally, to accommodate the attentional capacity of young children, we only included eighty trials in our study. If more trials were included in the current study, participants would have been provided with more opportunity to take part in hypothesis testing, and as a result a larger subset of children may have been able to adopt the correct strategy. Future research exploring category learning in young children may benefit from measuring categorization performance across a series of testing sessions, as not to deplete attentional resources.

Lastly, adults in the current study were all university students, which may not be the best representation of an adult population, limiting the extent to which we can generalize our findings to a general adult population. However, given the finding that 10–11 year-olds and adults performed quite similarly on the categorization task, our restricted age-range of adult participants is less concerning.

### Implications & Conclusions

The present findings have important implications for real-world categorization learning and training. When teaching tasks that involve complicated rules, providing children with working memory training may benefit performance. Holmes, Gathercole, and Dunning [37] have shown that providing children with adaptive training that taxed working memory to its limits was associated with sustained gains in working memory and improvements in mathematical ability. Based on findings from the current study showing that executive functioning may influence rule-based categorization performance, providing children with working memory/executive functioning training in future studies may result in improvements in category learning performance.

The current study examined rule-based category learning in children and adults. Rule-based categorization performance improved with age, with younger children struggling with the task, and older children approaching the performance of adults. Model based analyses helped to identify the type of strategy being used to solve the task. Results revealed that with age came increased use of the task appropriate strategy. Among participants using the task appropriate strategy, average categorization performance did not differ between children and adults. When accounting for executive functioning ability, working memory but not inhibitory control was a marginally significant partial mediator of the relationship between age and categorization performance. This suggests that age-related improvements in category learning may be in part explained by working memory capacity. Among children, rule-based category learning was associated with a larger forward digit span and better Flanker task performance. Interestingly, the use of the task appropriate strategy by children was associated with better working memory (as measured by the forward and backwards digit span) and better inhibitory control on some task measures (overall Flanker performance, and fewer Simon task errors). Furthermore, improvements in categorization performance may be explained, in part, by the executive functioning abilities of children ages 6–11.
